# Anti-Inflammatory Cytokines: Important Immunoregulatory Factors Contributing to Chemotherapy-Induced Gastrointestinal Mucositis

**DOI:** 10.1155/2012/490804

**Published:** 2012-09-02

**Authors:** Masooma Sultani, Andrea M. Stringer, Joanne M. Bowen, Rachel J. Gibson

**Affiliations:** ^1^School of Medical Sciences, University of Adelaide, North Terrace, Adelaide, SA 5005, Australia; ^2^School of Pharmacy and Medical Sciences, University of South Australia, North Terrace, Adelaide, SA 5001, Australia

## Abstract

“Mucositis” is the clinical term used to describe ulceration and damage of the mucous membranes of the entire gastrointestinal tract (GIT) following cytotoxic cancer chemotherapy and radiation therapy common symptoms include abdominal pain, bloating, diarrhoea, vomiting, and constipation resulting in both a significant clinical and financial burden. Chemotherapeutic drugs cause upregulation of stress response genes including *NF**κ**B,* that in turn upregulate the production of proinflammatory cytokines such as interleukin-1**β** (IL-1**β**), Interleukin-6 (IL-6), and tumour necrosis factor-**α** (TNF-**α**). These proinflammatory cytokines are responsible for initiating inflammation in response to tissue injury. Anti-inflammatory cytokines and specific cytokine inhibitors are also released to limit the sustained or excessive inflammatory reactions. In the past decade, intensive research has determined the role of proinflammatory cytokines in development of mucositis. However, a large gap remains in the knowledge of the role of anti-inflammatory cytokines in the setting of chemotherapy-induced mucositis. This critical paper will highlight current literature available relating to what is known regarding the development of mucositis, including the molecular mechanisms involved in inducing inflammation particularly with respect to the role of proinflammatory cytokines, as well as provide a detailed discussion of why it is essential to consider extensive research in the role of anti-inflammatory cytokines in chemotherapy-induced mucositis so that effective targeted treatment strategies can be developed.

## 1. Introduction

Cancer patients receiving chemotherapy and/or radiation therapy often develop mucositis as a direct result of their treatment. The term “mucositis” specifically refers to the damage of mucous membranes throughout the entire gastrointestinal tract (GIT) following chemotherapy and radiotherapy [1–3]. It is a major oncological problem reported in approximately 40% of patients undergoing standard dose chemotherapy and in almost all patients receiving high-dose chemotherapy and stem cell transplantation [4–7]. The prevalence of mucositis also varies depending on the type of cancer and therefore the combination of cytotoxic drugs [8, 9]. For example, patients treated with 5-fluorouracil (5-FU), often experience more severe mucositis [9]. Patients with mucositis exhibit severe clinical symptoms including pain due to ulceration of the GIT, nausea, vomiting, heartburn, diarrhoea, constipation, and therefore severe weight loss [1, 4, 7]. Furthermore, ulceration of the GIT is commonly associated with a high risk of systemic infection which poses a threat to patient health [10]. Mucositis can result in unplanned treatment interruptions including dosage reduction or premature cessation of cancer treatment [1, 3, 7]. Patients may require prolonged hospitalization and administration of antibiotic, antiviral therapy, or antifungal drugs depending on the severity of the condition [9, 10]. Currently, management of mucositis is largely supported with treatment limited to pain relief, maintenance of good oral hygiene, and the use of loperamide (a nonanalgesic opioid) to treat diarrhoea [11]. Hence, mucositis is a major clinical and economic burden that severely impacts patients' quality of life and increases their risk of morbidity and mortality [12]. Within the previous decade, intense research has clarified the complex sequence of molecular events underlying the pathobiology of mucositis and the development of novel treatments and strategies in the management of mucositis. 

## 2. Pathobiology of Mucositis

The pathobiology of mucositis is complex and involves the interplay of multiple intricate pathways including molecular and cellular events that occur in all layers of the gastrointestinal mucosa [13]. Historically, it was assumed that mucositis development was simply an epithelial phenomenon and occurred due to the direct toxic effect of cytotoxic chemotherapeutic agents on the basal cells of the gastrointestinal tract epithelium [9, 14, 15]. However, recent investigations have clearly identified that mucositis development is complicated with the involvement of molecular pathways between all mucosal compartments [8, 9, 15–17]. Initially, mucositis development was proposed in a four-stage model by Sonis in 1998 [18]. However subsequent studies have further extending this model into a five stage model comprising of the (1) *initiation*, (2) *upregulation and message generation*, (3) *signalling and amplification*, (4) *ulceration and inflammation*, and (5) *healing* phase [9, 14, 19, 20]. 

Briefly, the initiation phase occurs immediately following exposure to cytotoxic therapy resulting in direct damage to cellular DNA leading to immediate cell injury or death in the basal epithelia and submucosal cells [9, 13]. Furthermore, extensive tissue injury can result in the generation of reactive oxygen species (ROS). ROS are known to cause damage to cells and tissues, stimulate macrophages, and trigger a cascade of inflammatory pathways including the SP1-related retinoblastoma control protein, p53, and the nuclear factor kappa-B (NF-*κ*B) inflammatory pathway which is described in detail later in this paper [14, 21, 22].

The upregulation and message generation phase involves the activation of a number of signalling pathways and transcription factors, most importantly NF*κ*B [13, 23]. NF*κ*B in turn mediates gene expression and synthesis of various inflammatory molecules including proinflammatory cytokines such as tumour-necrosis factor (TNF), interleukin-6 (IL-6), interleukin-1*β* (IL-1*β*), adhesion molecules, and cyclooxygenase-2 (COX-2) from adjacent connective tissue [21, 22, 24]. The initiation of this inflammatory cascade has been confirmed in the clinical setting where studies have demonstrated the presence of these inflammatory factors in peripheral blood samples of chemotherapy patients [24, 25].

Signal amplification is the third phase of mucositis development where the inflammation signal is further amplified as a consequence of proinflammatory cytokines such as TNF acting in a positive feedback loop to reinforce NF*κ*B activation [13, 18, 21]. The net effect of this amplified inflammatory positive feedback loop is enhanced production of proinflammatory cytokines (TNF, IL-6, and IL-1*β*) and further tissue damage as a result of increased apoptosis.

Mucositis is clinically evident during the fourth ulcerative phase where the gastrointestinal epithelium integrity is destroyed. The GIT epithelium is characterised by the formation of pseudomembranes and ulcers [13, 15, 18, 21]. Bacterial colonisation at the site of ulceration further induces inflammation and activates the infiltration of macrophages and other inflammatory cells to the site of tissue injury [26].

The final healing phase occurs within approximately two weeks following cessation of treatment [21] and is a spontaneous self-resolving process where the intestinal epithelium is renewed. Although it remains the least understood stage of mucositis, it is thought that COX-2 may play an important “rebuilding” role in the healing phase by initiating angiogenesis which is predominantly seen in this phase [21, 27].

## 3. Inflammatory Signalling Pathways in Mucositis 

### 3.1. The Role of Cytokines in Tissue Inflammation

Cytokines are pleiotropic endogenous inflammatory and immunomodulating mediators that exhibit both negative and positive regulatory effects on various target cells [28–30]. These cell-derived polypeptides closely orchestrate both acute and chronic inflammatory processes by acting locally or systemically on the site of tissue infection via autocrine and paracrine pathways [30]. Briefly, inflammation at the site of infected tissue arises from the activation of various resident inflammatory cells such as fibroblasts, endothelial cells, tissue macrophages, and mast cells as well as the recruitment of monocytes, lymphocytes, and neutrophils [30]. This aggregation of inflammatory cells at the site of inflammation is initiated by a number of soluble mediators such as cytokines, inflammatory lipid metabolites such as platelet activating factor (PAF), and derivatives of arachidonic acid such as prostaglandins [30]. Such inflammatory effects can give rise to swelling due to fluid accumulation, increased blood flow and vascular permeability resulting in redness, and pain [30]. As inflammation closely correlates with the production of cytokines, inflammatory events that occur during mucositis development have also been thought to be associated with the generation of cytokine signalling cascade [29].

### 3.2. Nuclear Factor *κ*B (NF*κ*B): A “Gate-Keeper” Secondary Messenger

Evidence from previous literature clearly describes the role of nuclear factor *κ*B (NF*κ*B) transcription factor in the development of mucositis [8, 9, 31, 32]. NF*κ*B is a ubiquitous transcription factor that collectively comprises of the following five members: NF-*κ*B1 (p50/p105), NF-*κ*B2 (p52/p100), p65 (Rel A), Rel 3, and cRel [9, 33]. It typically resides as an inactive heterodimer of p65/RelA and p50 or p52 subunits bound to the members of I*κ*B (Inhibitor kappa B) proteins in the cell cytoplasm [32, 34]. Upon activation by an extensive range of agents such as bacteria and bacterial cell wall products, viruses, cytokines, free radicals scavengers from oxidative stress, ionizing radiation, and even the use of antineoplastic agents (cisplatin, doxorubicin, taxol, paclitaxel, and etoposide), NF*κ*B acts to induce gene expression of many cytokines involved predominantly in mucosal inflammation, and angiogenesis, chemokines, immunoreceptors, cell adhesion molecules, proapoptotic and antiapoptotic as well as stress response genes [9, 31–33]. On a molecular level, NF*κ*B stimulation signals cause the phosphorylation of NF*κ*B inhibitory protein I*κ*B, located in cell cytoplasm [35]. This results in the dissociation of bound NF*κ*B from I*κ*B which is subsequently translocated to the cell nucleus where it upregulates the expression of approximately 200 genes [8]. I*κ*B is further degraded by proteases [14]. 

In the past decade, much attention has been given to the diverse roles NF*κ*B plays in generating tissue response by targeting a wide range of genes [8, 34]. Its role in potentiating inflammatory and immune responses by inducing various proinflammatory cytokines production such as TNF, IL-6 and IL-1*β* involved in the development of mucositis has been widely recognised with the use of animal models and in the clinical setting [5, 34]. NF*κ*B is considered a key “driver” of chemotherapy-induced mucositis as its activation correlates with the production of TNF, IL-6 and IL-1*β* the hallmarks of mucositis inflammation [8, 36]. In particular, cytotoxic drug administration results in the upregulation of NF*κ*B and subsequently proinflammatory cytokine (TNF, IL-6, and IL-1*β*) levels [8, 37, 38]. In further support of this, Logan et al. (2008) reported a significant rise in serum NF*κ*B, TNF, IL-6, and IL-1*β* levels following administration of three different chemotherapeutic drugs known to cause mucositis [37]. Irinotecan, a commonly used cytotoxic agent, has also been shown to significantly elevate NF*κ*B production in the oral mucosa, jejunum, and colon [8, 34]. This elevation ultimately culminates in villus blunting, epithelial atrophy, and increased inflammatory cell infiltration in all tissues [8]. Further research clearly indicates that NF*κ*B activation is stimulated by chemotherapeutic agents thus leading to the production of proinflammatory cytokines, TNF, IL-6, and IL-1*β*, resulting in mucosal damage [8, 37, 38]. 

In support of the animal studies, clinical evidence is also available. Research from our group has further demonstrated a significant rise in tissue NF*κ*B levels in the oral buccal mucosa of cancer patients undergoing chemotherapy [34]. Yeoh et al. (2005) published data correlating histopathological changes with increased NF*κ*B and COX-2 expression in the colonic mucosa of patients treated with ionizing radiation [5]. In addition, ionizing radiation-induced NF*κ*B activation has been reported in other studies where radiation is known to cause the generation of oxygen free radicals, damage to cellular components, and breakage of the DNA double strands [39–41]. These inflammatory markers are essential in the pathogenesis of mucositis development and therefore in assessing the severity of tissue damage. 

## 4. Proinflammatory Cytokines and Inflammation

As described earlier, the activation of NF*κ*B results in the production of proinflammatory mediators such as TNF, IL-6, and IL-1*β* [8, 9, 18, 21, 28]. This subclass of cytokines is referred to as “*proinflammatory cytokines*” due to their ability to promote inflammation in response to tissue injury and infection [42]. Another subclass of cytokines is the “*anti-inflammatory cytokines*” which are involved in suppressing the activity of proinflammatory cytokines hence downregulating the inflammatory response [43]. Overexpression of anti-inflammatory cytokines is known to lead to depression of the immune system thus rendering the host at risk of systemic infection [43, 44]. Previous studies have directly implicated the presence of TNF, IL-6, and IL-1*β* proinflammatory cytokines in the pathogenesis of a number of inflammatory diseases, such as inflammatory bowel disease (IBD) [45], rheumatoid arthritis [46], sepsis [47], and most importantly, in mucositis [8, 13, 21, 34]. The role of proinflammatory cytokines has been discussed in detail in other critical reviews and is outside the scope of this paper. For further details please refer to Logan Review [8]. However the role of anti-inflammatory cytokines is discussed in detail below.

## 5. Anti-Inflammatory Cytokines and Inflammation

**Figure 1:**
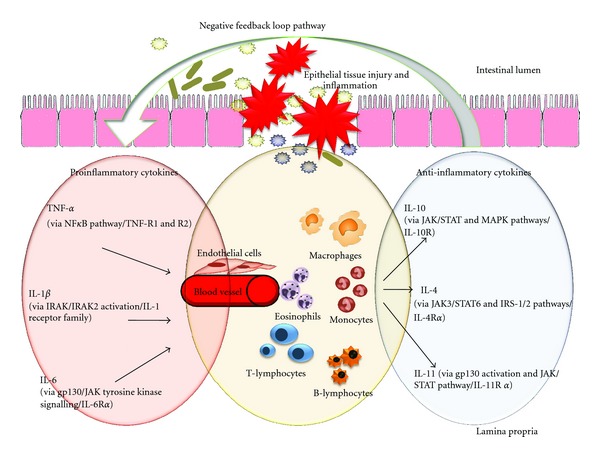
Possible negative feedback mechanism between pro- and anti-inflammatory cytokines IL-10, IL-4, and IL-11 following intestinal damage and inflammation.

Under normal physiological conditions, the human immune system comprises of multiple redundant pathways and immunoregulatory control elements that act in concert to coordinate the immune response initiated upon an external signal [43]. Of these multifaceted components of the immune system, the anti-inflammatory cytokine component has been subject to in-depth research for years. Studying the regulation of inflammation by these cytokine inhibitors is complicated as a number of related external factors must be considered for appropriate understanding and analysis of net effect of these cytokines. It has been postulated that factors such as timing of cytokine release, local environment in which it acts, presence of competing or synergistic elements, nature of target cells, availability and density of specific cytokine receptors, and tissue-specific response to each cytokine determine their net effect [43]. In addition, the discovery of various proinflammatory cytokines has further added to the complexity of the intricate pathways that occur during an immune response. Collective findings from a wide range of cytokine investigations indicate that the net effect of the inflammatory response is determined by a delicate balance between pro- and anti-inflammatory cytokines [43] as shown in Figure 1. Perturbations in this equilibrium can drive the host defence immune response either towards chronic inflammation or towards healing [43]. To date, various anti-inflammatory cytokines have been acknowledged in literature and these include IL-1ra, IL-4, IL-6, IL-10, IL-11, IL-13, TGF-*β*, and various soluble cytokine receptors [43]. This literature paper will address four of these anti-inflammatory cytokines in detail: IL-4, IL-10, IL-11, and IL-1ra. 

### 5.1. Interleukin-4 (IL-4)

IL-4 is a 20-kDa polypeptide secreted by mature Th-2 type helper T cells, mast cells, and basophils [48, 49]. IL-4 has marked inhibitory effects on the expression and release of proinflammatory cytokines [43]. Molecular and structural analysis of the IL-4 receptor has identified the 140-kDa IL-4R*α* chain where high-affinity binding with IL-4 occurs and further dimerization of this complex with the *γ*-chain mediates intracellular cell signalling [50, 51]. These cell surface IL-4 receptor complexes have been discovered in an extensive range of tissues including hematopoietic, endothelial, epithelial, muscle, fibroblast, hepatocytes, and brain tissues thus accounting for their broad range of activity [50].

IL-4 suppresses IL-1*β* synthesis, a major proinflammatory cytokine involved in inducing inflammation [52]. Furthermore, IL-4 enhances the expression of IL-1 receptor antagonist (IL-1ra), an antagonist that blocks the binding of proinflammatory cytokines, IL-1*α* and IL-1*β*, to their specific receptors [52, 53]. By mediating its action through the widely expressed IL-4 receptor *α* (IL-4R*α*), IL-4 plays an important role in tissue adhesion and inflammation as well as potentiates immunological effects against gram-negative bacterial infections [54, 55]. This highlights the diverse complex biological effects of IL-4 in mediating immunity through a unique array of cellular responses. Although evidence suggests that IL-4 has potential anti-inflammatory effects, its role during mucositis remains undefined. To date, there is no evidence to suggest whether IL-4 is produced at tissue levels in mucositis and if it is upregulated during different stages of mucositis. Thus it is obligatory for forthcoming studies to address these issues in order to develop better understanding of the anti-inflammatory properties of IL-4 during inflammation in mucositis.

### 5.2. Interleukin-10 (IL-10)

**Figure 2:**
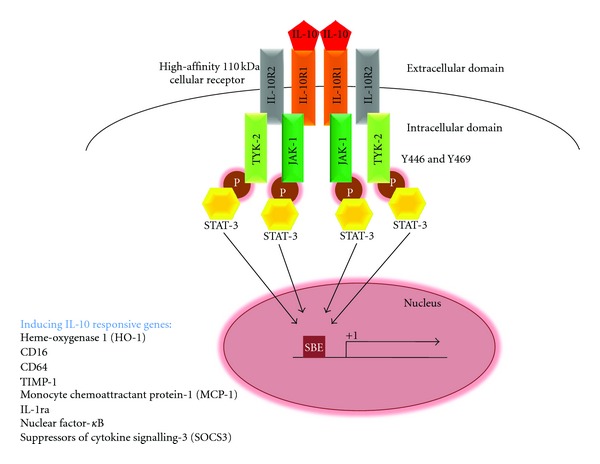
Diagram illustrating the JAK-STAT signal transduction pathway activated by anti-inflammatory cytokine, Interleukin-10. IL-10 binds its high-affinity extracellular IL-10 receptors in a homodimeric form which leads to intracellular receptor-associated Janus tyrosine kinases, JAK-1 and TYK-2. These activated kinases serve a temporary docking-sites for the cytosolic STAT-3 via their phosphorylated SH-2 domain. Activated STAT-3 translocates to the nucleus and binds to STAT Binding Element (SBE) to promote a wide range of IL-10 responsive genes.

IL-10 is the central anti-inflammatory cytokine well researched in the pathogenesis of Inflammatory Bowel Disease (IBD). Active IL-10 is secreted by CD4+ Th2 cell, Treg, monocyte, and macrophage cells of the immune system [56, 57].  Figure 2 shows the IL-10 receptor activation that induces a wide range of inflammatory controlling genes during tissue injury.

IL-10 controls inflammatory processes by suppressing the expression of proinflammatory cytokines, chemokines, adhesion molecules, as well as antigen-presenting and costimulatory molecules in monocytes/macrophages, neutrophils, and T cells [57, 58]. Early *in vitro* studies demonstrated IL-10 suppresses monocytes/macrophage-derived proinflammatory cytokines such as TNF-*α*, IL-1, IL-6, IL-8, and IL-12 [59, 60]. Additional studies support the notion that IL-10 attenuates TNF-receptor expression and further promotes its shedding into systemic circulation [61, 62]. Together these findings indicated IL-10 is an important immunoregulatory factor that significantly contributes to decreasing the intensity of inflammatory response by downregulating proinflammatory cytokine production at the site of tissue damage.

In an attempt to report the effect of IL-10 on NF*κ*B, *in vitro* analysis by Clarke and Colleagues (1998) showed that IL-10 is capable of inhibiting the activation of LPS-induced NF*κ*B in macrophages and pre-B cells [61]. This study supports the evidence that IL-10 mediates anti-inflammatory effects by inhibiting the up-stream NF*κ*B transcription factor, an essential secondary messenger required for inducing proinflammatory cytokine gene expression. 

In the pathogenesis of IBD, the potent immunosuppressive effects of IL-10 have been highlighted in several studies. The IL-10 knockout mouse model has successfully portrayed spontaneous development of chronic inflammatory enteritis, a condition similar to IBD in humans, suggesting that endogenous IL-10 is a central regulator of the mucosal immune response [63, 64]. Further dysregulation of the ratio of pro/anti-inflammatory cytokines, IL-1*β*/IL-1ra, has been associated with IL-10 administration in mucosal biopsies of UC patients [65]. In addition, *in vitro* analysis by Schreiber et al. (1995) also demonstrated that IL-10 downregulates the enhanced production of proinflammatory cytokines from IBD mononuclear phagocytes [66]. Thus, low production of IL-10 anti-inflammatory cytokine in the mucosa of IBD patients has been regarded as an important factor in the pathogenesis of IBD. Such data in the pathogenesis of mucositis lacks and investigation is warranted. Essentially recognised as an inflammatory condition, the highly complex and interactive nature of mucositis pathobiology strictly limits our approach towards targeting an appropriate molecular pathway. This evidence strongly supports the notion that IL-10 is in fact a crucial cytokine with anti-inflammatory properties that remains to be investigated in the setting of chemotherapy-induced mucositis. 

### 5.3. Interleukin-11 (IL-11)

IL-11 is a well-known pleiotropic cytokine. Physiological levels of IL-11 expression is identified in a wide range of normal adult murine tissues including thymus, spleen, bone marrow, heart, lung, small and large intestine, kidney, brain, testis, ovary, and uterus [67, 68]. IL-11 functions to control inflammation, ameliorate tissue damage, and maintain cytokine haemostasis during infection by acting on various cell types including hematopoietic precursor cells, macrophages, adipocytes, epithelial, and T cells [69, 70]. IL-11 mediates its biological activities by binding to its low affinity IL-11*α* receptor (IL-11R*α*) and subsequently complexing with the gp130 signal transduction subunit commonly shared with IL-6 receptor [70–72].

There is much evidence highlighting the anti-inflammatory properties of IL-11 in duration of an inflammatory response. Trepicchio et al. (1996) used mouse peritoneal macrophages and effectively demonstrated that rhIL-11 reduced the production of a wide spectrum of proinflammatory mediators such as TNF-*α*, IL-1*β*, IL-12, and NO [73]. Furthermore, administration of rhIL-11 in mice reduced proinflammatory cytokine production during a systemic inflammation [73]. In a follow-up study to elucidate the molecular mechanisms of IL-11 activity, *in vitro* analysis in LPS-stimulated peritoneal macrophages by Trepicchio et al. (1997) illustrated that rhIL-11 blocked nuclear translocation of NF*κ*B transcription factor and showed increase cytoplasmic levels of the NF*κ*B inhibitory proteins, I*κ*B-*α* and I*κ*B-*β* [74]. In addition, mRNA detection of the IL-11R*α* and gp130 complex in human and murine CD4^+^ and CD8^+^ lymphocytes suggests that IL-11 enhances the direct production of T-cell-derived anti-inflammatory cytokines such as IL-4 and IL-10 [76]. Together, these findings clearly establish the immunoregulatory pleiotropic properties of IL-11 in substantiating inflammatory responses during cytokine-derived tissue injury. 

Due to its potent anti-inflammatory nature, IL-11 has been under thorough investigation in several animal models exhibiting inflammatory conditions such as gut microorganism-induced sepsis [77], chronic IBD [78], and ischemic bowel necrosis [79]. As a multifunctional cytokine, IL-11 acts to attenuate the production of proinflammatory cytokine in correlation with this ability to reduce inflammation [75]. Flow cytometric analysis by Trepicchio and Colleagues revealed that administration of rhIL-11* in vitro *successfully attenuated the production of IL-1*β* and TNF-*α* from activated peritoneal macrophages [73]. Furthermore, rhIL-11 treatment also reduced serum levels of LPS-induced IFN-*γ*, a well-known proinflammatory cytokine that enhances inflammation through further activation of macrophages [73, 80].

In the context of gastrointestinal inflammation, IL-11 has been of particular interest due to its anti-inflammatory and mucosal protective effects. In a rat model of colitis, Peterson et al. (1998) demonstrated that rhIL-11 treatment ameliorated the development of colitis by downregulating the production of proinflammatory cytokines as well as maintaining the trophic structure of the gastrointestinal epithelium [81]. Recombinant human IL-11 enhances recovery from mucosal injury after cancer chemotherapy treatment. Gibson and Colleagues investigated the effect of IL-11 on ameliorating mucositis in a rat model implanted with syngeneic breast cancer following chemotherapy [49]. It was concluded that IL-11 has significant protective trophic effects on the intestinal epithelium as chemotherapy-related damage of the villous atrophy and crypt length damage was less severe. Also, IL-11 did not produce protective effects on the breast cancer tissue in this rat model of mucositis further highlighting its efficacy and safety upon administrating an effective dose [49]. In 1995, Sonis and Colleagues primarily established that IL-11 decreases the severity and duration of mucosal inflammation by protecting the intestinal epithelium and connective tissue using a hamster model of oral mucositis [82]. Liu et al. (1996) also demonstrated that IL-11 significantly increased villus height and the rate of crypt cell mitosis in the rat model of short bowel syndrome [83]. The IL-11 receptor-*α* (IL-11R*α*) is expressed within the gastrointestinal epithelium and colonic epithelial cells of the mucosa. *In vitro* studies have confirmed that IL-11 directly interacts on untransformed IEC-18 epithelial cells to inhibit cellular proliferation [84, 85]. Although IL-11 is well researched in a number of different inflammatory conditions, current literature lacks to understand the direct mechanism of IL-11 on the intestinal epithelium relating to ameliorating chemotherapy-induced mucositis. 

### 5.4. Interleukin-1 Receptor Antagonist (IL-1ra)

IL-1 receptor antagonist is a naturally synthesised and secreted 23–25-kDa glycosylated protein produced primarily by monocytes, macrophages, neutrophils, microglial cells, hepatocytes, and many other cells in response to tissue injury, infection, and inflammation [86, 87]. 

Extensive molecular research has established the central biological role of IL-1ra as a highly competitive antagonist of its functional proinflammatory ligands, IL-1*α* and IL-1*β*. For many years, these isoforms of the IL-1 cytokine family have been recognised to participate in initiating and amplifying inflammation upon induced tissue injury and infection. Amongst its various pleiotropic localised and systemic effects, IL-1 cytokines are known to promote inflammatory cell infiltration at site of tissue injury, induce fever and vascular dilation, promote NO, COX-2, and prostaglandin E_2_ production, and induce production of other cytokine mediators such as IL-6 [88].

Evidence from previous independent studies have identified that IL-1 cytokines, in particular IL-1*β*, play crucial roles in the pathogenesis of various gastrointestinal tract associated inflammatory conditions such as IBD, ischemic-reperfusion injury, chronic enteritis, and irritable bowel syndrome (IBS) [89].

An *in vitro* study by Al-Sadi and Ma (2007) has additionally highlighted the role of IL-1*β* on gastrointestinal tract epithelial obliteration by successfully showing that at a dose of 10 ng/mL, IL-1*β* effectively increased tight-cell junction permeability in the Caco-2 cell line 48 hours following treatment [89]. This data provides strong evidence that elevated IL-1*β* levels during intestinal injury further amplifies inflammation by disrupting epithelial barrier and increasing paracellular permeation of toxic luminal agents into the mucosa [89]. 

Furthermore, Andus et al. (1997) proposed a lower IL-1ra to IL-1 ratio in inflamed mucosa samples from patients with Crohn's Disease (CD) highlighting the importance of localised tissue IL-1ra presence to downregulate excessive inflammation [90]. IL-1ra animal models have also been utilised in previous investigations to establish a clear understanding of the role of IL-1ra during inflammation. *IL-1ra* gene knockout mice are known to be highly susceptible to suffering from endotoxemia and develop spontaneous joint inflammation, arthritis thus resulting growth deficit [91].

In recent years, scientific research has shifted focus towards determining the anti-inflammatory role of IL-1ra during mucositis development. Preliminary microarray data provided by Wu et al. (2011) identified the *IL-1ra* gene to be highly expressed in correlation with increased serum levels of IL-1ra after 5-FU treatment [92]. In a tumour-bearing mouse model of 5-FU-induced mucositis, exogenous application of IL-1ra significantly reduced intestinal crypt cell apoptosis and severity of diarrhoea without affecting 5-FU-induced tumour regression [93]. Further comprehensive analysis of the role of IL-1ra is necessary to validate its protective anti-inflammatory effects in the context of chemotherapy-induced mucositis prior to its application in a clinical setting. 

## 6. Tregs and Cancer

Regulatory T cells, more commonly referred to as Tregs, are responsible for the induction and maintenance of peripheral tolerance, a critically important function enabling the body to suppress immune responses by influencing different cell type activity [94]. Tregs make up approximately 10% of thymus-derived CD4+ T cells, coexpressing the CD25 antigen (IL-2R *α*-chain), and requiring Fox3p transcription factor expression for suppressive phenotype [95]. Two main types of Tregs exist, natural and peripherally-induced, with natural Tregs primarily responsible for controlling immune responses to autoantigens [94], and induced Tregs inhibiting inflammation [94, 96]. Both types of Tregs contribute to overall tumour tolerance in many cancers including, but not limited to pancreatic, [97] ovarian [98], melanoma [99], and renal cell carcinoma [96, 100]. Given the importance of Tregs in tumour tolerance, Tregs, have gained increased recognition in cancer patients and treatments. Wolf and Colleagues (2003) examined the Treg levels in peripheral blood samples of 42 cancer patients and 34 healthy controls and found cancer patients had increased numbers of Tregs compared with controls. Importantly, these increases were associated with immunosuppression. The authors suggested that the increase in Tregs in cancer patients may negatively impact the effectiveness of immunotherapies including monoclonal antibody therapy [101]. 

Most relevant to this paper, one of the immune suppressive mechanisms by which Tregs maintain immune homeostasis is through secretion of the anti-inflammatory cytokines IL-10 and TGF*β* [102]. Importantly, IL10 is required to maintain immune homeostasis in the gut, where Treg-specific deletion of IL-10 leads to colitis in mice [103]. Since evidence shows that Th1 immune responses simultaneously stimulate Treg induction [102], prevention of inflammation should theoretically be an effective means of limiting Treg-produced anti-inflammatory cytokines.

### 6.1. Therapy Options for Tregs

A number of clinical studies have been reported in recent years examining the potential use of Tregs as a specific marker of treatment response in a number of different cancer types. Kaufman and Colleagues conducted a small clinical study to examine the effectiveness of interleukin-2 on Treg responses in metastatic renal cell carcinoma. They enrolled 25 patients and found that although Tregs were elevated in all patients prior to treatment, in patients who achieved stable disease state a 50% reduction in the Tregs was seen. These findings suggest that interleukin-2 therapy is effective in reducing Tregs, although the study was extremely small [100]. These findings are in agreement with an earlier clinical study, whereby 11 patients with renal cell carcinoma vaccinated with DAB_389_IL-2 (recombinant IL-2 diphtheria toxin) had their Tregs eliminated from peripheral cells [95]. 

Ascierto et al. [99] evaluated the Treg levels in 22 melanoma patients following IFN-a 2b therapy and reported that prior to therapy, Treg levels were significantly higher (*P* < 0.001) in melanoma patients compared to healthy controls. Following therapy, Treg levels decreased although this did not reach significance. The authors suggested that it was impossible to draw any conclusions regarding Tregs as a marker to cancer treatment response due to small sample size [99].

There are many other papers which suggest a variety of ways in which Tregs can be “managed” in the context of cancer. These include histone deacetylase inhibitors (which would augment the suppressive function of Tregs) [104], retinoids [105], Dendritic cell vaccines [106], and blocking either Treg effector functions [107] or Treg differentiation [108]. This list is by no means exhaustive, but a more detailed analysis is beyond the scope of this paper. 

## 7. Conclusions

In the past decade, active research has been conducted to define the pathogenesis of mucositis and the role of proinflammatory cytokines TNF-*α*, IL-6, and IL-1*β*. Evidence of the upregulation of these proinflammatory cytokines coordinated with the extent of mucosal injury in mucositis proves to be valuable. This subclass of cytokines is recognised to play an enormous role during inflammation and tissue damage in response to cytotoxic therapy. There remains, however, a huge gap in the knowledge to recognise whether anti-inflammatory cytokines such as IL-4, IL-10, IL-11, and IL-1ra are essential tools in downregulating the inflammatory response associated with mucositis. Lack of this knowledge which ties pro- and anti-inflammatory cytokines together within the complex yet interesting cytokine milieu leaves an incomplete image of immune response associated with mucositis. Furthermore, there is no evidence in literature that interprets the net balance of the subclass of cytokines in accordance with different phases of mucositis development. Moreover, the underlying mechanisms of action of these anti-inflammatory cytokines in chemotherapy-induced mucositis remain underresearched. Taking into consideration the lack of knowledge of anti-inflammatory cytokines in the setting of chemotherapy-induced mucositis, it is obligatory for future cytokine studies of inflammation to base their research on identifying and interpreting the interrelationships of anti-inflammatory cytokines in the pathogenesis of mucositis.
